# Editorial: Mechanisms and Novel Therapies in Graves’ Orbitopathy: Current Update

**DOI:** 10.3389/fendo.2022.902591

**Published:** 2022-04-29

**Authors:** Huifang Zhou, Ilaria Muller, Kelvin Kam-Lung Chong, Marian Ludgate, Sijie Fang

**Affiliations:** ^1^ Department of Ophthalmology, Shanghai Ninth People’s Hospital, Shanghai JiaoTong University School of Medicine, Shanghai, China; ^2^ Shanghai Key Laboratory of Orbital Diseases and Ocular Oncology, Shanghai Ninth People’s Hospital, Shanghai JiaoTong University School of Medicine, Shanghai, China; ^3^ Department of Clinical Sciences and Community Health, University of Milan, Milan, Italy; ^4^ Department of Endocrinology, Fondazione Istituto di Ricovero e Cura a Carattere Scientifico (IRCCS) Ca’ Granda Ospedale Maggiore Policlinico, Milan, Italy; ^5^ Division of Infection and Immunity, Cardiff University School of Medicine, Cardiff, United Kingdom; ^6^ Department of Ophthalmology and Visual Sciences, The Chinese University of Hong Kong, Hong Kong, Hong Kong SAR, China

**Keywords:** graves orbitopathy, epidemiology, pathogenesis, evaluation, novel therapeutic approach

Graves’ orbitopathy (GO) is the main extra-thyroidal manifestation in patients with Graves’ disease (GD), with a prevalence of up to 50%, although in most cases GO is mild (1). GO has a variety of clinical manifestations including eyelid retraction, exophthalmos, restrictive strabismus, exposure keratitis, and dysthyroid optic neuropathy. It is the commonest orbital disease causing blindness, orbital deformity and visual disability and exerts a profound negative impact on patients’ quality of life (2).

This Research Topic provides a timely update on different aspects of GO, in 20 excellent contributions, ranging from epidemiology, pathogenesis, disease evaluation, to comments on existing therapies and novel treatment strategies. Bartalena et al. reviewed GO incidence and prevalence, then highlighted risk factors such as age and smoking, and how they might be regulated or avoided to optimize clinical management. Clinically, hyperthyroidism and orbitopathy often develop simultaneously or within a few months of each other (2). Both thyroid epithelia and orbital fibroblasts (OF) express the thyrotropin receptor (TSHR), suggesting that these two conditions may evolve from common underlying systemic processes (1). Uncontrolled hyperthyroidism, which is caused by autoantibodies (TRAB) to the TSHR in GD, is a major GO risk factor with high TRAB levels long known to correlate with GO incidence and severity (3). In this context, George et al. provided a comprehensive overview introducing thyroid-stimulating antibody (TSAb) as a biomarker for GO whilst Kelada et al. investigated polyautoimmunity as a risk factor of GO activity and severity from their retrospective cohort of 267 GO patients.

The pathogenesis of GO is complex and not fully understood although some consensus has emerged in recent years (4). Most would regard the OFs as target cells contributing to the tissue remodeling which leads to expansion of the orbital contents. Draman et al. summarized OF signaling cascades and how they affect two of these processes, adipogenesis and hyaluronan production. To further our understanding of OFs, Virakul et al. compared the proteome of DNA methylation of OFs from GO patients and healthy controls. They found over-expression of genes implicated in inflammation and proliferation, together with subtle differences between active and inactive GO profiles. A similar microarray approach was applied to the GO lacrimal gland by Tu et al.

GO is an autoimmune disease and orbital inflammation is initiated by the loss of self-tolerance to the putative, shared antigens between thyroid glands and orbits, notably the TSHR (5). Antigen-presenting cells (APC) recognize and present TSHR to T helper (Th) cells for unknown reasons, and the existence of a soluble TSHR may be implicated (6). Upon antigen activation, T cells interact physically with APCs and facilitate B cell proliferation and differentiation, ultimately leading to autoantibody production. It is widely accepted that both cellular and humoral immunities contribute to the GO pathogenesis (3, 7). Some studies have suggested the importance of Th1 cells in the early active phase and Th2 cells in the chronic fibrotic phase of GO (7). An increased number of Th17 cells have been detected at the site of orbital connective tissues (8, 9), leading to the concept of an imbalance in effector and regulatory T cells in GO autoimmunity. Fang et al. comprehensively summarized the current knowledge on T cell immunity in GO: Th1 (cytotoxic leaning), Th2 (antibody leaning) and an emerging role of Th17 (fibrotic leaning) cell subsets, in the context of key pathogenic processes such as adipogenesis and fibrosis. Hypothesizing that auto-immune responses can be locally triggered and aggravated, Lu et al. characterized the T cell repertoire infiltrating paranasal sinus mucosae in GO patients and reported increased pro-inflammatory effector T cells but reduced regulatory T cells.

Despite advances in understanding, the current treatment for GO is not satisfactory and long-term deformities and disabilities often persist. The problem is exacerbated by the fact that GO presentation can be heterogenous, as highlighted by an overview of asymmetric GO by Panagiotou and Perros, focusing on its clinical relevance and possible mechanisms. The Amsterdam declaration recommends that GO patients should be managed in joint endocrinology-ophthalmology clinics (10). An interesting study from Farag et al. described a ‘real world’ snapshot of the multidisciplinary management of a multi-ethnic GO population prior to the introduction of established GO standards.

Several existing therapies are currently used in the management of GO. In light of the inflammatory and immunological processes in operation, the first-line treatment is the administration of intravenous glucocorticoids (11). Naselli et al. reported that GO patients with high levels of low-density lipoproteins cholesterol were likely to respond poorly to glucocorticoids. In mild GO, selenium was shown to be beneficial, *via* its anti-oxidant and immunomodulatory effects, as reviewed by Lanzolla et al. The effects of anti-B cell therapies are somewhat controversial but indicate an effective role of rituximab for early active moderate-to-severe GO (12, 13). Campi et al. performed a *post-hoc* analysis on the efficacy of rituximab in GO patients, which can be helpful in the decision-making process. In addition, Zhang et al. described the salvaging benefits of rituximab in 2 patients refractory to glucocorticoids who subsequently underwent orbital decompression.

In order to develop novel effective target therapies for GO, it is essential to clarify the key pathological mechanisms, both immune and non-immune related, occurring within the orbit. The concept of circulating bone marrow derived CD34^+^ fibrocytes in the pathogenesis of GO is original and impactful. These TSHR-expressing progenitor cells migrate from the peripheral blood into orbital connective tissues and transit into CD34^+^ OFs that upon stimulation, undergo adipocytic and myofibroblastic differentiation (14). TSHR signaling in fibrocytes and OFs are partially dependent on the insulin-like growth factor 1 receptor (IGF-1R), another putative autoantigen that has received increasing attention in the past few years (15). Years of *in vitro* research were recently translated into a phase 2 and then a phase 3 randomized multicenter trials which consistently demonstrated that GO patients treated with teprotumumab, a fully human monoclonal IGF-1R inhibitor, were significantly more likely to experience a meaningful improvement in proptosis compared with patients treated with placebo (16, 17). Smith, one of the main researchers involved, reviewed the encouraging findings of teprotumumab as the first targeted therapy for GO. The cross-talk between TSHR and IGF-1R mediated by PKA/PI3K-FOXO signaling highlights a feasible therapeutic strategy to attenuate signaling initiated at either receptor, thereby relieving GO processes (4, 18).

Traditional immunosuppressive agents such as mycophenolate, azathioprine, and cyclosporin mainly inhibit the activation and proliferation of T cells (4). To date, no therapy targeting a particular T cell subset has been reported. Fortunately, blocking T cell related cytokines (e.g. IL-6) such as tocilizumab shows promising results in GO (19). Fallahi et al. from Antonelli’s group shared their thoughts on cytokine-based therapy in GD and GO.

Previous studies reported changes in T cell subsets during the transition from hyperthyroidism to euthyroidism and from active to inactive GO (20, 21). Thus, whilst GD and GO shared similar antibody-mediated immune attack, as highlighted by the improvement of these conditions in a patient with thyroid cancer treated with a TSHR-blocking monoclonal antibody (22), cell-mediated immunity is believed to play a central role in GO pathogenesis. A phase 1 multicenter trial revealed that ATX-GD-59, a mixture of two TSHR-derived peptides binding with HLA-DR on dendritic cells, improved free thyroid hormone levels in 70% (7/10 responders) of previously untreated mild to moderate Graves’ hyperthyroidism (23).

*In vitro* studies have facilitated identification of other novel treatments: Lanzolla et al. reviewed the use of antioxidant agents in mild GO; Wei et al. proposed simvastatin and ROCK inhibitors for orbital fibrosis whilst Lee et al. demonstrated potential for proprotein convertase as therapeutic target and biomarker.

The field of GO research has benefitted from the development of a robust TSHR-induced *in vivo* model of GO (24, 25). Comparison of the gut microbiota composition between mice housed in 2 centers revealed significant differences which may account for heterogeneity in the induced immune responses. When diseased and control mice were compared, disease-associated taxonomies were identified e.g. *Firmicutes* positively correlated with orbital adipogenesis (26). The same researchers modified the gut microbiota using antibiotic, probiotic and human fecal material transfer (hFMT). Experimental GO was exacerbated by hFMT and ameliorated by antibiotic treatment confirming a role for the gut microbiome in GD/GO (27). Several groups have investigated whether the same applies in human GD/GO as reviewed by Wang et al., who also provide a comprehensive summary of epigenetic and other factors implicated in the GO disease process.

In conclusion, this Research Topic provides encouraging updates made in understanding the complex interactions between genetic background and environmental factors such as the gut microbiome. It reviews the efficacies and short-comings of emerging therapies (e.g. teprotumumab and potential hearing problems) which have evolved from years of translational research to date. It illustrates an unlimited potential of non-surgical treatments, many of which target fundamental disease processes such as ligand-receptor binding and T cell subset imbalance underpinning GO development, and will ultimately benefit patient care in the near future.

## Author Contributions

SF and HZ wrote the paper. ML, IM, and KC revised the paper. ML was the senior author of the paper. All authors contributed to the article and approved the submitted version.

## Funding

This work was supported by the National Natural Science Foundation of China (81930024, 82071003, 82000879) and the Research Grant of the Shanghai Science and Technology Committee (20DZ2270800).

## Conflict of Interest

The authors declare that the research was conducted in the absence of any commercial or financial relationships that could be construed as a potential conflict of interest.

## Publisher’s Note

All claims expressed in this article are solely those of the authors and do not necessarily represent those of their affiliated organizations, or those of the publisher, the editors and the reviewers. Any product that may be evaluated in this article, or claim that may be made by its manufacturer, is not guaranteed or endorsed by the publisher.
